# Exploring the clinical utility of angioinvasion markers in papillary thyroid cancer: a literature review

**DOI:** 10.3389/fendo.2023.1261860

**Published:** 2023-11-27

**Authors:** Angelika Buczyńska, Maria Kościuszko, Adam Jacek Krętowski, Anna Popławska-Kita

**Affiliations:** ^1^Clinical Research Centre, Medical University of Bialystok, Bialystok, Poland; ^2^Department of Endocrinology, Diabetology and Internal Medicine, Medical University of Bialystok, Bialystok, Poland

**Keywords:** papillary thyroid cancer, angioinvasion, VFGF, oxidative stress, integrins, sortilin, podoplanin

## Abstract

Papillary thyroid cancer (PTC) is the most common type of thyroid cancer, and angioinvasion, the invasion of blood vessels by cancer cells, is a crucial pathological feature associated with disease progression and poor prognosis. Thus, a comprehensive search of scientific databases was conducted to identify relevant studies investigating angioinvasion markers in PTC. The selected studies were reviewed and analyzed to assess the clinical significance and potential utility of these markers in predicting angioinvasion and guiding treatment decisions. Numerous studies have investigated various markers associated with angioinvasion in PTC, including oxidative stress, vascular endothelial growth factor (VEGF), matrix metalloproteinases (MMPs), and other angiogenic factors. The results indicate that increased expression of these markers is correlated with the presence and extent of angioinvasion in PTC. Moreover, some studies suggest that these markers can serve as prognostic indicators and guide therapeutic strategies, such as selecting patients for more aggressive treatment approaches or targeted therapies. The findings from the reviewed literature highlight the potential clinical utility of angioinvasion markers in PTC. The identification and validation of reliable markers can aid in assessing the risk of angioinvasion, predicting disease progression, and optimizing treatment decisions for patients with PTC. However, further research and validation on larger patient cohorts are necessary to establish the robustness and generalizability of these markers in clinical practice.

## 1 Introduction

Papillary thyroid cancer (PTC) is the most prevalent type of thyroid malignancy, accounting for approximately 80% of all thyroid cancer cases (1). Although generally associated with a favorable prognosis, PTC can exhibit varying degrees of aggressiveness, with certain cases demonstrating increased potential for invasion and metastasis (2). Angioinvasion, the invasion of cancer cells into blood or lymphatic vessels, is a critical determinant of tumor behavior and can significantly impact patient outcomes. Accurate assessment of angioinvasion in PTC is crucial for predicting disease progression, selecting appropriate treatment strategies, and optimizing patient management. While traditional histopathological techniques, such as lymphovascular invasion evaluation, have been utilized to assess angioinvasion, the identification of specific molecular markers has the potential to enhance diagnostic precision and prognostic accuracy (3).

Nowadays, various molecular markers have been investigated for their potential association with angioinvasion in PTC. Notably, vascular endothelial growth factor (VEGF), a well-known pro-angiogenic factor, has shown correlations with angioinvasion and tumor aggressiveness (4). Additionally, CD34, a cell surface glycoprotein used as a marker of microvessel density, and podoplanin, a protein involved in lymphatic vessel formation, have been implicated in angioinvasion in PTC (5, 6). Among others, oxidative stress markers, sortilin and integrins have been implicated in angiogenesis and may contribute to the angioinvasive phenotype of PTC (2). Understanding the molecular mechanisms underlying angioinvasion in PTC is crucial for improving diagnostic accuracy, prognostication, and therapeutic decision-making (7).

Through a comprehensive review of the existing literature, this study aims to consolidate the current knowledge on angioinvasion markers in PTC and explore promising novel markers. The study aimed to identify potential markers that can reliably indicate the presence of angioinvasion in PTC, which can improve risk stratification and facilitate personalized therapeutic interventions for patients with PTC.

## 2 Materials and methods

This literature review employed a systematic approach to identify and analyze studies investigating novel angioinvasion markers in PTC. The methodology ensured the inclusion of relevant studies and provided a rigorous evaluation of the available literature in this field (8). Thus, a systematic search was performed to identify relevant articles from various electronic databases, including PubMed, Scopus, and Web of Science (9). The search strategy utilized a combination of keywords and controlled vocabulary terms related to PTC, angioinvasion, and molecular markers. The search was conducted with no restrictions on language or publication date. Articles were included if they met the following criteria: PTC, investigated angioinvasion markers, provided relevant data or findings, and were published in peer-reviewed journals. Studies that were reviews, editorials, or conference abstracts were excluded. Full-text articles of the selected studies were then assessed for eligibility. Data from the included articles were extracted using a standardized form. The extracted data included study characteristics (e.g., author, year of publication), patient population, study design, angioinvasion markers investigated, methodology, and key findings. The extracted data were analyzed thematically to identify common trends, associations, and novel markers implicated in angioinvasion in PTC. The findings were summarized and presented descriptively. The quality and risk of bias of the included studies were evaluated using appropriate tools, such as the Newcastle-Ottawa Scale for observational studies (10) or the Cochrane Collaboration’s tool for randomized controlled trials (11). As this study is a literature review, ethical approval was not required. The data were obtained from published studies, ensuring confidentiality and anonymity of the participants. The limitations of this study included the potential for publication bias, as only peer-reviewed articles were included, and the reliance on available literature. Additionally, the heterogeneity of the included studies may have affected the ability to perform quantitative analysis.

## 3 Papillary thyroid cancer epidemiology

PTC is the most common type of thyroid cancer, accounting for approximately 80-90% of all thyroid malignancies (12). The incidence of PTC has been steadily increasing over the past few decades in many countries worldwide (13, 14). This rise in incidence is partly attributed to increased detection due to advanced imaging techniques and improved diagnostic practices. Furthermore, PTC exhibits a strong female predominance. Women are approximately three times more likely to develop PTC than men (15). This gender disparity has been observed consistently across different populations and geographic regions. Several studies have investigated potential hormonal (estrogen receptors have been found in thyroid tissue, and estrogen can stimulate the growth and proliferation of thyroid cells) and genetic factors (such as BRAF mutation, other genetic alterations, such as RET/PTC rearrangements and RAS mutations) contributing to this gender difference (16). Moreover, PTC can affect individuals of all age groups, but it is most commonly diagnosed in adults aged 30-50 years. Thus, the incidence of PTC has been increasing among younger populations, particularly adolescents and young adults. The reasons for this age-specific trend are not fully understood, and further research is needed to explore potential risk factors (15). Nevertheless, PTC incidence rates vary significantly across different countries and regions. Higher incidence rates have been reported in regions with a higher iodine intake, such as areas of high goiter prevalence (17). The impact of environmental factors, genetic predisposition, and variations in healthcare practices on these geographical differences is a subject of ongoing research (17). Interestingly, exposure to ionizing radiation, especially during childhood, is a well-established risk factor for PTC (18). Individuals who have undergone radiation therapy for medical conditions, such as Hodgkin’s lymphoma or head and neck cancers, have an increased risk of developing PTC (19). Studies have consistently shown a dose-response relationship between radiation exposure and PTC risk.

The study conducted by Baloch et al. aimed to identify prognostic factors in well-differentiated follicular-derived carcinoma. It investigated various clinicopathological factors to determine their impact on patient prognosis. The results of the study revealed several significant prognostic factors associated with PTC prognosis, including age, tumor size, presence of lymph node metastasis, extrathyroidal extension, and distant metastasis. Older age, larger tumor size, presence of lymph node metastasis, and extrathyroidal extension were correlated with a poorer prognosis, while the presence of distant metastasis indicated a significantly worse outcome. The study provided valuable insights into prognostic factors, such as angioinvasion measurement, that can help predict the outcomes of patients with well-differentiated follicular-derived carcinoma, specifically PTC. These findings emphasize the importance of clinicians making informed decisions regarding treatment strategies and patient management based on angioinvasion detection and the presence of lymph node metastasis. Ultimately, angioinvasion detection could lead to improved patient care and outcomes (20).

## 4 Clinical management of PTC

The clinical management of PTC involves a multidisciplinary approach that includes surgery, radioactive iodine (RAI) therapy, thyroid hormone replacement, and long-term follow-up (21). The specific management strategy may vary depending on factors such as tumor characteristics, stage, patient age, and angioinvasion with lymph metastasis detection (22). The primary treatment for PTC is most frequently thyroidectomy. The extent of surgery may vary from a total thyroidectomy to a lobectomy (23). Lymph node dissection may be performed if there is evidence of lymph node involvement. The goal of surgery is to remove the primary tumor and any involved lymph nodes while minimizing the risk of recurrence. Following thyroidectomy, RAI therapy may be recommended for certain PTC patients, particularly those with high-risk features such as larger tumors, angioinvasion presents, lymph node involvement, or distant metastasis (24). RAI therapy involves the administration of a radioactive iodine isotope (iodine-131) that selectively targets and destroys any remaining thyroid tissue or cancer cells. This adjuvant therapy aims to eliminate residual disease and reduce the risk of recurrence (25). After thyroidectomy, lifelong thyroid hormone replacement therapy is essential to achieve suppression of thyroid-stimulating hormone (TSH) levels and to maintain normal thyroid hormone levels. This involves daily oral intake of synthetic thyroid hormone (levothyroxine) to replace the natural thyroid hormone produced by the thyroid gland (26). The goal is to suppress TSH levels, which helps prevent tumor growth and recurrence. Regular monitoring of thyroid hormone levels and adjustment of medication dosage is necessary to ensure optimal hormone replacement (27). Thus, PTC patients require long-term follow-up care to monitor for disease recurrence, assess treatment response, and manage any potential complications (28). In cases of locally advanced or metastatic PTC that does not respond to standard treatments, additional therapeutic options may be considered (29). These may include targeted therapies, such as tyrosine kinase inhibitors (e.g., lenvatinib, sorafenib), which block specific molecular pathways involved in cancer growth and angiogenesis. Clinical trials investigating novel therapies may also be an option for eligible patients (30, 31). From the other hand, in low-risk PTC less invasive techniques could be use. Thermal ablation refers to the use of heat-based techniques to treat thyroid nodules, including PTC. It is a minimally invasive procedure that aims to destroy or shrink the tumor without the need for surgery (32). Thermal ablation has shown promising results in the treatment of PTC, with studies demonstrating effective tumor control and minimal complications (33). However, it is important to note that surgery, such as thyroidectomy and lobectomy, remains the primary treatment approach for most cases of PTC (34). Therefore, identifying the presence or absence of angioinvasion would allow avoiding invasive procedures and the need for therapy for a large group of patients, as well as the requirement for lifelong observation. Furthermore, it would help identify a subgroup of patients at high risk for more aggressive progression of PTC, where the introduction of more radical therapy would be recommended as soon as possible.

## 5 Angioinvasion: markers and clinical implications

The evaluation of angioinvasion markers in PTC typically involves the assessment of specific molecular and cellular markers associated with tumor angiogenesis and invasiveness. These markers may include Vascular Endothelial Growth Factor (VEGF), CD34, podoplanin, integrins, and matrix metalloproteinases (MMPs) and oxidative stress markers. Through various techniques such as immunohistochemistry, gene expression analysis, and biomolecular assays, the expression levels and presence of these markers can be quantified and correlated with angioinvasion in PTC (3).

Accurate evaluation of angioinvasion markers holds significant clinical implications. It can provide valuable information regarding tumor behavior, likelihood of lymph node metastasis, risk of recurrence, and overall patient prognosis (35). This information can guide treatment decisions, such as the extent of surgery (total thyroidectomy vs. lobectomy), the need for lymph node dissection, and the consideration of adjuvant therapies. Furthermore, the identification and validation of novel angioinvasion markers in PTC have the potential to revolutionize clinical treatment options. Targeted therapies that specifically inhibit angiogenesis and tumor invasiveness could be developed, leading to improved patient outcomes (36). These therapies may include anti-angiogenic agents, tyrosine kinase inhibitors, or other molecular targeted therapies aimed at disrupting the signaling pathways involved in angiogenesis and tumor progression (37). However, it is important to note that while several markers have shown promise in preclinical and early clinical studies, further research is needed to validate their clinical utility, establish standardized diagnostic criteria, and assess their effectiveness in larger patient populations (38). Rigorous clinical trials and translational research efforts are required to determine the efficacy, safety, and long-term outcomes of targeting angioinvasion markers in PTC treatment. Nevertheless, the evaluation of angioinvasion markers among PTC patients is essential for accurate prognosis determination and treatment decision-making (39). Continued research into novel markers and the development of targeted therapies hold great potential for improving clinical outcomes and personalized treatment options for PTC patients (40).

## 6 Oxidative stress involvement in PTC angioinvasion

Oxidative stress is known to play a role in the angioinvasion of PTC. Oxidative stress refers to an imbalance between the production of reactive oxygen species (ROS) and the body’s antioxidant defense mechanisms, leading to cellular damage (41). Several studies have indicated that oxidative stress is involved in promoting angiogenesis, which is the process of new blood vessel formation that facilitates tumor growth and metastasis (42). In PTC, oxidative stress can arise from various sources, including increased ROS production by tumor cells, inflammation, and altered antioxidant capacity (43). The excessive production of ROS can induce the activation of signaling pathways that promote angiogenesis. These pathways involve factors such as VEGF, hypoxia-inducible factor 1-alpha (HIF-1α), and nuclear factor-kappa B (NF-κB), which contribute to the formation of new blood vessels in the tumor microenvironment (44). Furthermore, oxidative stress can lead to DNA damage and genetic alterations in tumor cells, promoting their invasive properties. It can also influence the expression of matrix metalloproteinases (MMPs), enzymes involved in extracellular matrix degradation and tumor invasion (45). MMPs play a crucial role in facilitating the breakdown of basement membranes and blood vessel walls, allowing tumor cells to invade surrounding tissues and enter the bloodstream (46).

The study performed by Azouzi et al. concerned on the role of nicotinamide adenine dinucleotide phosphate oxidase 4 (NOX4) as a critical mediator of BRAFV600E-induced downregulation of the sodium/iodide symporter (NIS) in PTC aimed to understand the mechanism underlying the loss of NIS expression in this disease. The results of the study demonstrated that activation of the BRAFV600E pathway led to upregulation of NOX4 expression, which in turn resulted in the overproduction of ROS in thyroid cancer cells. The increased ROS levels induced oxidative stress, leading to a decrease in NIS expression. It was found that NOX4 is directly involved in this process, and its inhibition restored NIS expression in thyroid cancer cells. Furthermore, it was observed that in tissue samples obtained from patients with papillary thyroid carcinoma, the presence of BRAFV600E and NOX4 correlated with decreased NIS expression. These findings highlight the significant role of NOX4 as a mediator of NIS downregulation in BRAFV600E-mutated thyroid cancer. These discoveries have important clinical implications as the loss of NIS expression hinders the effective use of radioactive iodine therapy. Understanding the mechanism regulating NIS loss may contribute to the development of new therapies aimed at restoring NIS function and enhancing the effectiveness of treatment for iodine-refractory thyroid cancer (47). The following study performed by Weyemi et al. concerned on the intracellular expression of the enzyme NOX4, a generator of ROS, in normal and cancer thyroid tissues aimed to investigate the role of NOX4 in the pathogenesis of thyroid cancer. The results of the study showed that NOX4 was expressed in both normal and cancerous thyroid tissues. However, the expression level of NOX4 was significantly higher in cancerous tissues compared to healthy tissue. It was also observed that NOX4 expression correlated with the presence of the transcription factor HIF-1α (hypoxia-inducible factor 1α) in thyroid cancer cells. HIF-1α is a known regulator of metabolic processes and the response to hypoxia. Additionally, it was observed that a high level of NOX4 expression was associated with more advanced clinical stages of thyroid tumors. This suggests a potential role of NOX4 in the progression and aggressiveness of thyroid cancer. These findings suggest that NOX4 may play a significant role in redox processes and the pathogenesis of thyroid cancer. Increased NOX4 expression in cancerous tissues may lead to the overproduction of ROS, which in turn can influence cell proliferation, angiogenesis, and other processes related to tumor development. Despite promising results, further research is needed to better understand the mechanisms regulating NOX4 expression and its role in the pathogenesis of thyroid cancer. This may lead to the identification of NOX4 as a potential therapeutic target or prognostic biomarker in this disease (48). Another study conducted by Mseddi et al. evaluating the nuclear 8-hydroxyguanosine (8-OHdG) expression in autoimmune thyroid diseases and PTC, and its relationship with cancer-related proteins p53, Bcl-2, and Ki-67 aimed to investigate potential differences in the level of oxidative DNA damage between these two disease states. The results of the study showed that nuclear 8-OHdG expression was increased in both autoimmune thyroid diseases and PTC compared to healthy thyroid tissue. However, significant differences in the expression level were observed between these two patient groups. In the case of PTC, a higher level of 8-OHdG expression was observed compared to autoimmune thyroid diseases. Additionally, a significant correlation was found between 8-OHdG expression and the cancer-related proteins p53, Bcl-2, and Ki-67 in the group of PTC patients. Higher levels of 8-OHdG expression were associated with higher levels of p53 and Ki-67, which are markers of cell proliferation, and lower levels of Bcl-2 protein, which is associated with apoptosis. The conclusions from this study suggest that oxidative DNA damage, represented by 8-OHdG expression, is present in both autoimmune thyroid diseases and PTC. However, differences in expression levels and associations with cancer-related proteins indicate potentially different mechanisms of oxidative stress in these two disease states. Further research is needed to better understand the role of oxidative DNA damage in autoimmune thyroid diseases and PTC, as well as the potential use of 8-OHdG as a diagnostic or prognostic biomarker in these diseases (49).

Understanding the involvement of oxidative stress in PTC angioinvasion is essential for developing targeted therapies (35). Strategies aimed at reducing oxidative stress or inhibiting specific molecular pathways associated with angiogenesis and invasion may hold promise for inhibiting tumor progression and improving patient outcomes (25). However, further research is needed to fully elucidate the mechanisms by which oxidative stress contributes to PTC angioinvasion and to identify potential therapeutic targets.

### 6.1 VEGF

#### 6.1.1 Angioinvasion biomarker

VEGF is a potent angiogenic factor that promotes the formation of new blood vessels. In the study conducted by Wreesmann et al., evaluating PTC tissues obtained from 47 patients with angioinvasive PTC, immunohistochemistry confirmed that increased VEGF expression is associated with angioinvasion. Moreover, high levels of VEGF have been correlated with more aggressive tumor behavior and poorer clinical outcomes (50). Several studies have also demonstrated the prognostic significance of VEGF expression in PTC. In the following study performed by Selemetjev et al., elevated VEGF expression has been associated with more aggressive tumor behavior, including larger tumor size, lymph node metastasis, and higher tumor (51). This study was also conducted on PTC tissues collected from 29 angioinvasive PTC. Additionally, higher VEGF expression levels have shown poorer clinical outcomes, including increased rates of tumor recurrence and decreased disease-free survival (52).

#### 6.1.2 VEGF inhibitor: novel clinical trails

Due to its role in promoting angiogenesis and tumor progression, VEGF has been explored as a potential therapeutic target in PTC. Inhibition of VEGF signaling pathways, either through targeted therapies or anti-angiogenic agents, has shown promising results in preclinical studies and clinical trials (53). These approaches aim to disrupt angiogenesis and inhibit tumor growth by targeting VEGF and its receptors. Firstly, in a phase III clinical trial (DECISION trial), sorafenib, a multi-kinase inhibitor that targets VEGF receptors, was evaluated in patients with locally advanced or metastatic radioactive iodine-refractory differentiated thyroid cancer (DTC). The study demonstrated that sorafenib significantly prolonged progression-free survival compared to placebo, leading to its approval by the US Food and Drug Administration (FDA) for the treatment of this patient population (54). Vandetanib is another multi-kinase inhibitor with activity against VEGF receptors. In a phase III clinical trial (ZETA trial), vandetanib was studied in patients with unresectable locally advanced or metastatic medullary thyroid cancer (MTC). The study demonstrated prolonged progression-free survival in the vandetanib-treated group compared to placebo (55). The following medicament, the Lenvatinib is a multi-kinase inhibitor with potent VEGF receptor inhibition. In a phase Ib/II clinical trial, the combination of lenvatinib and pembrolizumab (a PD-1 immune checkpoint inhibitor) was investigated in patients with advanced thyroid cancer, including both differentiated and medullary thyroid cancer. The study demonstrated promising antitumor activity, with a high response rate and manageable safety profile (56). The subsequent drug, apatinib is a small molecule inhibitor of receptors for vascular endothelial growth factor type 2 (VEGFR-2). Angiogenesis inhibition by blocking the VEGFR-2 is an emerging strategy to develop selective and specific anticancer agents. A case report described a patient with radioiodine-refractory papillary thyroid carcinoma who received apatinib treatment. The patient showed a significant reduction in tumor size and improvement in disease symptoms, suggesting potential efficacy in this setting (57).

#### 6.1.3 VEGF inhibitors clinical challenges

While VEGF inhibitors have shown effectiveness in PTC, the response rates may vary among patients. Not all patients will experience significant tumor shrinkage or prolonged survival (58). Identifying patients who are more likely to benefit from VEGF inhibition and understanding the mechanisms underlying treatment response are ongoing areas of research. Furthermore, VEGF inhibitors can cause side effects, including hypertension, proteinuria, bleeding, wound healing complications, and gastrointestinal perforation (30). Managing these toxicities and balancing the benefits and risks of treatment can be challenging for healthcare providers. Like other targeted therapies, resistance can develop over time in response to VEGF inhibition (59). Tumors can acquire genetic alterations or activate alternative signaling pathways that allow them to bypass VEGF dependency and continue growing. This can lead to treatment failure and disease progression. Thus, combining VEGF inhibitors with other treatment modalities, such as chemotherapy or immune checkpoint inhibitors, is an active area of investigation (60). However, determining the optimal sequencing, dosing, and duration of combination therapies can be complex (60). Clinical trials are ongoing to explore the potential synergistic effects and overcome resistance. Clearly, identifying reliable biomarkers that can predict response to VEGF inhibition in PTC is crucial for optimizing patient selection and treatment strategies. Currently, there are no established biomarkers that reliably predict response to VEGF inhibitors in PTC (36). Further research is needed to identify molecular markers or genetic alterations associated with treatment response.

### 6.2 Podoplanin

Podoplanin (PDPN) is a transmembrane glycoprotein that plays a crucial role in several physiological and pathological processes, including tumor progression and metastasis. Studies have consistently demonstrated that increased expression of podoplanin is associated with angioinvasion in various types of cancers, including PTC. Angioinvasion refers to the invasion of tumor cells into the blood or lymphatic vessels, which is closely correlated with tumor aggressiveness and the potential for metastasis (61). Numerous studies have suggested that podoplanin may contribute to lymphatic vessel formation and lymphatic invasion, which are closely associated with the process of angioinvasion in PTC. PDPN is predominantly expressed on the surface of lymphatic endothelial cells and is involved in regulating their functions. It interacts with its receptor, C-type lectin-like receptor 2 (CLEC-2), to promote lymphangiogenesis and enhance lymphatic vessel integrity. In PTC, elevated levels of PDPN expression have been detected in tumors exhibiting lymphatic invasion as compared to those without. This observation suggests that podoplanin could potentially serve as a marker to identify PTC cases that are more likely to have angioinvasion and a more aggressive clinical course (5). Additionally, studies have shown that high podoplanin expression is associated with adverse clinicopathological features and poorer outcomes in PTC patients. In the study performed by Sikorska et al. podoplanin was promoting aggressive phenotypes in PTC through the epithelial-mesenchymal transition (EMT) signaling pathway, which is associated with increased invasiveness and metastasis in cancer cells (6). This study also revealed that the impact of podoplanin on cell phenotype was influenced by the genetic background of thyroid tumor cells. Specifically, it has been observed that down-regulation of PDPN in BcPAP cells, a commonly used cell line derived from PTC, is associated with reduced migration and invasion capabilities. This suggests that PDPN plays a role in promoting the migration and invasiveness of BcPAP cells. On the other hand, in TPC1 cells, depletion of PDPN leads to increased migration and invasiveness. These findings highlight the potential importance of PDPN in regulating the migratory and invasive properties of cancer cells and suggest that its expression levels may impact the aggressive behavior of PTC cells. Furthermore, our findings suggest that PDPN may be involved in the epithelial-mesenchymal transition (EMT) process in BcPAP cells through its regulation of the expression of ezrin, radixin, and moesin (E/R/M) proteins, matrix metalloproteinases (MMPs) 9 and MMP2, as well as the remodeling of actin cytoskeleton and cellular protrusions (6). In the following study performed by Sun et al., examines the contribution of podoplanin-positive cancer-associated fibroblasts (CAFs) to the invasiveness of squamous cell carcinoma of the thyroid (62). In this study differences in the expression of cancer-associated fibroblast (CAF)-related proteins were observed in both cancer cells and stromal cells of PTC. These differences were found to vary based on histologic subtype, presence of the BRAF V600E mutation. Importantly, the expression of PDPN was associated with prognosis, suggesting their potential as prognostic markers in PTC. Moreover, the study performed by Lin et al., suggests that the characterization of circulating epithelial cells (CECs) with PDPN assessment holds promise as a diagnostic and prognostic tool in thyroid cancer. (63). In this study, the researchers performed molecular analysis of CECs and identified specific genetic alterations, such as mutations or gene expression changes, that were associated with aggressive tumor characteristics and worse prognosis.

These studies collectively suggest that the expression of PDPN may be associated with angioinvasion and tumor aggressiveness in PTC. Additional studies with larger sample sizes and standardized methodologies are necessary to further explore the potential of podoplanin in predicting disease progression and guiding treatment decisions in PTC.

### 6.3 Integrins

Integrins are a large family of cell surface receptors that play a crucial role in various biological processes, including cell adhesion, migration, and signaling. They are transmembrane proteins composed of α and β subunits, and their binding to specific ligands in the extracellular matrix or on other cells enables cells to adhere to their surroundings and interact with the extracellular environment. In addition to their adhesive and migratory functions, integrins are involved in signal transduction pathways (44). Binding of ligands to integrins triggers intracellular signaling cascades, leading to various cellular responses such as changes in gene expression, cytoskeletal reorganization, and modulation of cell proliferation and survival. Specific integrins, including αvβ3 integrin, have been found to play important roles in angiogenesis and tumor invasion in various types of cancer. αvβ3 integrin, in particular, has been studied extensively for its involvement in promoting the formation of new blood vessels and facilitating tumor cell invasion into surrounding tissues. Studies have suggested a possible association between αvβ3 integrin expression and angioinvasion in PTC, indicating its potential as a marker for assessing tumor aggressiveness. The study conducted by Arslan et al. aimed to examine the expression of integrin alpha-3 and beta-1 receptors in tumor tissue, metastatic lymph nodes, and normal tissue in thyroid cancer. The results of the study revealed the presence of integrin alpha-3 and beta-1 receptors in thyroid tumor tissue (64). This finding suggests that integrin may play a significant role in the development and progression of thyroid cancer. Additionally, integrin receptors were also present on metastatic lymph nodes, indicating their potential involvement in the process of tumor metastasis. Comparing to healthy tissue, the expression of integrin receptors was significantly higher on tumor tissue and metastatic lymph nodes. This study may have important clinical implications, as the identification of integrin receptors on thyroid tumor tissue could help in the development of new therapies targeting these receptors (64). Another study conducted by Liang et al. aimed to assess the utility of integrin αvβ3-targeted imaging using 99mTc-3PRGD2 in predicting disease progression in patients with high-risk differentiated thyroid cancer. 99mTc-3PRGD2 is a novel SPECT tracer specifically targeting the integrin α(V)β(3) receptor, which is involved in tumor detection and imaging angiogenesis. The study’s results indicated that integrin αvβ3-targeted imaging using 99mTc-3PRGD2 could serve as a promising method for predicting disease progression in these patients. The study observed that higher accumulations of 99mTc-3PRGD2 on scintigraphic images were associated with an increased risk of disease progression, including disease recurrence, metastasis, or worsening patient condition. Patients with greater accumulations of 99mTc-3PRGD2 exhibited poorer prognosis and shorter progression-free survival (65, 66). The subsequent study conducted by Mautone et al. aimed to investigate the correlation between the expression of integrin alpha 3 beta 1 receptors and patient outcomes in PTC. The study’s findings demonstrated that higher levels of integrin alpha 3 beta 1 receptor expression were associated with unfavorable outcomes in PTC. Patients with elevated expression of this receptor exhibited an increased risk of disease recurrence, metastasis, and overall prognosis deterioration. Patients with increased expression of this receptor exhibited a higher risk of disease recurrence, metastasis, and overall deterioration in prognosis. The conclusions drawn from this study carry significant clinical implications, as the identification of the level of expression of the integrin alpha 3 beta 1 receptor can assist in prognosis determination and decision-making regarding treatment (67).

The study conducted by Li et al. aimed to investigate the role of integrin β4 in PTC invasion and resistance to anoikis, a form of cell death triggered by loss of contact with the extracellular matrix. Additionally, the expression of integrin β4 in lymphovascular tumor thrombus was evaluated. The results of the study revealed that integrin β4 promotes invasion and resistance to anoikis in PTC. Elevated levels of integrin β4 were associated with an increased ability of cancer cells to breach the tissue barrier and invade surrounding tissues. Moreover, cancer cells with high expression of integrin β4 displayed heightened resistance to cell death caused by loss of contact with the extracellular matrix, thereby influencing their survival capacity and potential to form metastases. The study also demonstrated consistent overexpression of integrin β4 in lymphovascular tumor thrombus, which represents the spread of the tumor to blood and/or lymphatic vessels. This observation suggests that integrin β4 may play a significant role in tumor metastasis by enhancing cancer cell invasion and their ability to survive within the tumor microenvironment. The findings from this study underscore the potential of integrin β4 as an important prognostic and therapeutic factor in PTC. Inhibition of integrin β4 expression or function has the potential to curtail tumor invasion and augment the effectiveness of anticancer therapies (68, 69).

Cheng et al. conducted a study to explore the effects of blocking RGD-binding integrin activity in PTC cells. They found that inhibiting RGD-binding integrin had a significant impact on PTC cell behavior, including reduced migration, invasion, adhesion, and impaired blood vessel formation. Additionally, blocking RGD-binding integrin activity inhibited the AKT/mTOR signaling pathway, which is crucial for cancer cell growth, survival, and migration. These findings suggest that targeting RGD-binding integrin could be a promising therapeutic approach for PTC, as it can hinder disease progression, suppress invasive behavior, and impede new blood vessel formation necessary for tumor growth. The study highlights RGD-binding integrin as a potential therapeutic target in PTC. By inhibiting its activity, it may be possible to disrupt key cellular processes involved in tumor progression and angiogenesis. This research opens doors for novel strategies to combat PTC and improve treatment outcomes (70).

### 6.4 CD34

CD34 is a cell surface glycoprotein expressed by endothelial cells, commonly used as a marker to assess microvessel density and angiogenesis. The study by Majchrzak et al. investigated the prognostic value of angiogenesis markers, including CD31, CD34, and relative cerebral blood volume (rCBV), in low-grade gliomas. The results showed that higher expression levels of CD31 and CD34 were associated with increased vascularity and angiogenesis in low-grade gliomas. Higher rCBV values also correlated with increased angiogenesis. Moreover, patients with higher expression levels of CD31, CD34, and rCBV had a worse prognosis, indicating a link between increased angiogenesis and more aggressive tumor behavior (71). Another study by Fiedler et al. confirmed CD34 as a marker for lymphatic endothelial cells in human tumors, with its expression observed mainly in lymphatic vessels rather than blood vessels. The density of CD34-positive lymphatic vessels varied among tumor types and was associated with lymph node metastasis and poorer prognosis. CD34 is a cell surface glycoprotein that is not commonly expressed in normal thyroid tissue. However, its expression can be detected in certain types of thyroid cancer, particularly in poorly differentiated and anaplastic thyroid carcinomas (72). Studies have shown that CD34 expression in thyroid cancer is associated with more aggressive tumor behavior, including increased invasiveness, higher rates of metastasis, and poorer prognosis. The presence of CD34-positive blood vessels within the tumor microenvironment indicates angiogenesis, which is a crucial process for tumor growth and spread (73, 74).

In summary, these studies establish CD34 as a reliable marker for assessing angiogenesis and lymphatic vessels in human tumors, providing valuable prognostic information.

### 6.5 Matrix metalloproteinases and their tissue inhibitors

MMPs are a family of enzymes involved in the breakdown and remodeling of the extracellular matrix, which is a complex network of proteins and carbohydrates that provide structural support to tissues. MMPs play a crucial role in various physiological and pathological processes, including tissue development, wound healing, and cancer progression (75). MPPs and tissue inhibitors of metalloproteinases (TIMPs) have also been studied in relation to angioinvasion in PTC. MMPs are enzymes involved in extracellular matrix degradation, and their dysregulation can facilitate tumor invasion and metastasis. TIMPs act as inhibitors of MMPs and play a regulatory role in tumor progression. The study performed by Ivković et al. analyzed tissue samples from patients with PTC and assessed the expression levels of various MMPs, including MMP-2, MMP-9, and MMP-14, as well as their inhibitors, such as tissue inhibitors of metalloproteinases (TIMPs), specifically TIMP-1 and TIMP-2. The results of the study revealed that PTC tissues exhibited significantly higher expression levels of MMP-2, MMP-9, and MMP-14 compared to adjacent non-tumor tissues. This suggests that these MMPs are involved in the invasive nature of PTC, facilitating the degradation of extracellular matrix components and promoting tumor invasion. Additionally, the study found that the expression levels of TIMP-1 and TIMP-2 were reduced in PTC tissues compared to non-tumor tissues. This imbalance between MMPs and their inhibitors indicates a disrupted regulation of proteolytic activity in PTC, potentially contributing to the invasive phenotype of the cancer cells. Furthermore, the study observed a correlation between the expression levels of MMPs and their inhibitors with clinicopathological features of PTC. Higher expression levels of MMP-2, MMP-9, and MMP-14 were associated with advanced tumor stage, lymph node metastasis, and poorer prognosis. Conversely, lower expression levels of TIMP-1 and TIMP-2 were also associated with adverse clinicopathological parameters. These findings suggest that the dysregulation of MMPs and their inhibitors plays a significant role in the invasive behavior of PTC. The upregulation of MMPs and downregulation of TIMPs create an imbalance that promotes tumor invasion and metastasis. Thus, targeting MMPs or restoring the balance between MMPs and TIMPs could potentially serve as a therapeutic strategy to inhibit the invasive growth of PTC (76). Precisely, the study performed by Marečko et al. aimed to investigate the role of matrix MMP-9 in the infiltration and aggressiveness of PTC. The researchers analyzed tissue samples from patients with PTC and evaluated the expression and activity of MMP-9. They also examined the correlation between MMP-9 activation and the degree of tumor infiltration based on histopathological analysis. The results of the study demonstrated that MMP-9 expression and activation were significantly higher in PTC tissues compared to adjacent non-cancerous tissues. Moreover, there was a positive correlation between the degree of tumor infiltration and the level of MMP-9 activation. This suggests that increased MMP-9 activity is associated with more aggressive infiltration of PTC. Further analysis revealed that MMP-9 was predominantly localized in the tumor stroma and surrounding blood vessels. The researchers also observed a correlation between MMP-9 activation and the presence of lymph node metastasis, indicating its potential role in PTC metastasis. The findings of this study indicate that MMP-9 plays a crucial role in the infiltration and aggressiveness of PTC. Its enhanced activation is associated with a higher degree of tumor infiltration and lymph node metastasis. These results highlight the importance of MMP-9 as a potential therapeutic target and a prognostic marker for PTC (77). The following study conducted by Shi et al. investigate the potential of serum matrix metalloproteinase-2 (MMP-2) as a predictive marker for PTC. The researchers sought to determine whether the levels of MMP-2 in the serum of PTC patients could serve as a reliable indicator of the presence and progression of the disease. The researchers examined the relationship between MMP-2 levels and clinicopathological characteristics of PTC, including tumor size, lymph node involvement, and distant metastasis. The results of the study revealed that the serum levels of MMP-2 were significantly higher in PTC patients compared to the control group. This suggests that MMP-2 is involved in the pathogenesis of PTC and its elevation in the serum may reflect the presence of the disease. Furthermore, the study found that higher MMP-2 levels were associated with larger tumor size, lymph node metastasis, and distant metastasis. This indicates that elevated MMP-2 in the serum may serve as an indicator of more aggressive and advanced stages of PTC. Importantly, the researchers assessed the predictive value of serum MMP-2 levels in distinguishing PTC patients from individuals without thyroid abnormalities. They found that MMP-2 demonstrated good sensitivity and specificity in differentiating PTC patients from the control group, suggesting its potential as a predictive marker for PTC. Its elevated levels in the serum of PTC patients are associated with more advanced disease and could aid in the diagnosis and assessment of disease progression. However, further research and validation studies are needed to establish the clinical utility and reliability of serum MMP-2 as a predictive marker for PTC (78). These studies highlight the potential of MMPs assessment during TC clinical management especially tissue concentration of MMP-9 and serum MMP-2 considering as a predictive marker for PTC.

### 6.6 Sortilin

Sortilin, a protein involved in cellular trafficking and signaling, has been implicated in various aspects of cancer development and progression, including cancerogenesis (79). Research studies have shed light on the role of Sortilin in different types of cancers and have explored its potential as a diagnostic biomarker and therapeutic target. Studies investigating Sortilin in cancerogenesis have shown that its expression is often dysregulated in cancer cells compared to normal cells (80). Aberrant Sortilin expression has been observed in several types of cancers, including breast cancer, lung cancer, pancreatic cancer, colorectal cancer, and neuroblastoma, among others (81–83).

The exact role of Sortilin in cancerogenesis is still being elucidated, but evidence suggests its involvement in key processes such as cell proliferation, survival, migration, invasion, and angiogenesis (84). Sortilin has been found to interact with various ligands, including growth factors, neurotrophins, and extracellular matrix proteins, contributing to cancer cell behavior and tumor progression (85). Furthermore, studies have suggested that Sortilin may modulate signaling pathways that are crucial for cancerogenesis, especially in PTC pathogenesis, such as the PI3K/Akt pathway, MAPK/ERK pathway, and Wnt signaling pathway (86). These pathways regulate cell growth, differentiation, and survival, and dysregulation of these pathways is frequently associated with cancer development. Thus, the study performed by Faulkner et al. investigated the expression and potential therapeutic targeting of neurotrophin receptors in thyroid cancer. The researchers found that neurotrophin receptors TrkA, p75NTR, and Sortilin were significantly increased in thyroid cancer tissues compared to normal thyroid tissues. This suggests that these receptors may play a role in the development and progression of thyroid cancer. Furthermore, the study demonstrated that targeting these neurotrophin receptors with specific inhibitors or antibodies could inhibit the growth and survival of thyroid cancer cells. This finding suggests that these receptors could serve as potential therapeutic targets for thyroid cancer treatment. The results of this study provide valuable insights into the molecular mechanisms involved in thyroid cancer and highlight the potential for developing targeted therapies against neurotrophin receptors in this disease (86).

## 7 Conclusions

The evaluation of angioinvasion markers among papillary thyroid cancer (PTC) patients plays a crucial role in determining prognosis and guiding treatment decisions. When many studies have investigated the role of oxidative stress and VEGF in association with PTC angiogenesis, several factors have never been validated in clinical management. Thus, to confirm the role of VEGF as a marker of angiogenesis in PTC and to evaluate the clinical significance of new markers, such as MMP-2, Sortilin and CD34 further research and validation studies on larger patient populations are necessary. Studies on these factors can provide a more comprehensive understanding of angiogenesis in PTC and enable the identification of patients who may require more aggressive clinical management. The investigation of angiogenesis markers in PTC has also enabled the identification of medical targets in more advanced types of PTC, which form the basis for new clinical studies on PTC treatment.

## Author contributions

AB: conceptualization, data curation, investigation, software, validation, visualization, writing – original draft. MK: formal analysis, investigation, writing – original draft. AK: methodology, supervision, validation, writing – review & editing. AP-K: conceptualization, funding acquisition, resources, validation, visualization, writing – review & editing.
